# Co-designing Behavior Change Resources With Treatment-Seeking Smokers: Engagement Events' Findings

**DOI:** 10.3389/fpubh.2021.555449

**Published:** 2021-03-15

**Authors:** Nadia Minian, Mathangee Lingam, Wayne K. deRuiter, Rosa Dragonetti, Peter Selby

**Affiliations:** ^1^Nicotine Dependence Service, Centre for Addiction and Mental Health, Toronto, ON, Canada; ^2^Department of Family and Community Medicine, University of Toronto, Toronto, ON, Canada; ^3^Campbell Family Mental Health Research Institute, Toronto, ON, Canada; ^4^Institute of Medical Science, Faculty of Medicine, University of Toronto, Toronto, ON, Canada; ^5^Department of Psychiatry, University of Toronto, Toronto, ON, Canada; ^6^Dalla Lana School of Public Health, University of Toronto, Toronto, ON, Canada

**Keywords:** engagement event, co-design, smoking cessation, diet, alcohol, stress, behavior change interventions, physical activity

## Abstract

**Background:** Primary care organizations are well-suited to help patients change their unhealthy behaviors. Evidence shows that risk communication and self-monitoring of behavior are is an effective strategy practitioners can use to promote health behavior change with their patients. In order for this evidence to be actionable, it is important to understand how patients would like this information to be communicated and to operationalize the self-monitoring resources. The objective of this study was to co-create resources that encourage behavior change based on the scientific evidence and from patients with lived experiences.

**Materials and Methods:** Twenty-seven individuals who participated in a smoking cessation program and engaged in at least one other unhealthy behavior joined one of two engagement events. Each event was 3 h in duration and consisted of two exercises that provided support to participants in reaching a consensus about the types of messages they would like to receive from their practitioner as well as self-monitoring resources they would prefer to use. The first exercise followed an adapted version of the Consensus Methodology developed by the Institute of Cultural Affairs Canada, while the second exercise was in accordance to the Nominal Group Technique.

**Results:** Participants' preference was to have practitioners convey messages to promote health behavior change that include positive affirmation and to monitor all their health behaviors using a single self-reported tracking sheet.

**Conclusions:** This paper features the use of engagement events to reflect upon and identify potential resources that treatment seeking smokers prefer to receive while attempting to modify unhealthy behaviors. These resources can be used by health care providers in primary care settings to support health promotion interventions and assist their patients to increase their likelihood of adopting positive changes to risk behaviors.

## Introduction

Behaviors, such as excessive alcohol consumption, physical inactivity, and poor diet, are associated with an increased risk of mortality from numerous chronic conditions including cardiovascular disease, cancer, and stroke (1–6). In Canada, as well as globally, the development of strategies to reduce the prevalence of multiple unhealthy behaviors that are scientifically rigorous and patient-oriented is long overdue.

Although policy level interventions that address root causes of the behavior are most effective (7–10), for some individuals, clinical interventions may also be necessary for successful behavior change to be achieved (11–13). There is substantial evidence showing that when health care providers address health behaviors with their patients they can have a significant effect on their patient's efforts to achieve smoking cessation (14, 15), reduce harmful alcohol consumption (16), increase exercise (17), as well as attain positive changes in diet (18), mood (19), stress (20), and sleep (21, 22).

Behavior change techniques (BCTs) are considered to be the smallest active ingredient of an intervention that work to promote change in an individual (23). BCTs are theorized to operate by either enhancing factors that can facilitate the behavior change or by minimize the factors that would typically inhibit the behavior change (24). In 2010, a systematic review of behavior change techniques found that, relative to other techniques, “risk communication” and “self-monitoring of behavior” were effective strategies practitioners can use to promote health behavior change with their patients (25). It has been postulated that self-monitoring BCT works by allowing users to regulate their behavior, specifically encouraging behavioral, and/or cognitive skills for managing or changing behavior (24). Within Michie et al.'s BCT taxonomy (23) these two techniques fall within “1. Goals and Planning” specifically “1.6 Discrepancy between current behavior and goal” (for risk communication) and “2. Feedback and Monitoring,” specifically “2.3 Self-monitoring of behavior” (for self-monitoring) (23) However, while the benefits of self-monitoring are well-established (26, 27), the effectiveness of risk communication is not as clear; a recent systematic review of reviews, found that providing risk communication by itself does not lead to behavior changes that are sustained over time (28).

To make these research findings more meaningful and actionable, there is a need to contextualize these findings in the lives of people with lived experience. Including the perspectives of people with lived experience is known to improve the effectiveness and value of the programs aimed at improving population health (29–31). Given that our plan was to embed a behavior change resource into an Ontario-wide smoking cessation program, called the Smoking Treatment for Ontario Patients (STOP) program, we wanted to understand the perspective of STOP participants. Specifically how health care providers should communicate the need to change behaviors (including exploring the need for risk communication) and what self-monitoring resources they would use.

The STOP program is an established smoking cessation program implemented in primary care settings across Ontario, Canada, which offers up to 26 weeks of smoking cessation treatment, consisting of nicotine replacement therapy and behavioral counseling, at no cost to the patient. In addition to smoking, over 90% of STOP participants self-report two or more unhealthy behaviors. In January 2019, with funding from the Public Health Agency of Canada (PHAC) and the Medical Psychiatry Alliance (MPA), a new initiative called Picking Up the PACE (Promoting and Accelerating Change through Empowerment) was introduced into the STOP program to support practitioners address modifiable risk factors (e.g., physical inactivity, poor diet) with their patients. Based on the results of two co-creation events, Picking Up the PACE developed an online tool that encourages practitioners to (1) Communicate to patients the need for health behavior change and (2) Provide self-monitoring resources. The co-creation of the tools and messages allows users to have a voice on how health promotion programs and products are designed and offered. This is important given that there is promising evidence that participatory approaches to health promotion, that accounted for patient-identified priorities, ultimately lead to better patient outcomes (32).

This manuscript describes the methodology we used to co-create health promotion tools (self-monitoring worksheets and messages health care providers can use to communicate with their patients the need to change their behaviors). We used the guiding principles outlined in the Strategy for Patient-Oriented Research Patient Engagement Framework (32), namely inclusiveness, support, mutual respect, and co-building. We combined these with the recommendations that emerged from co-creation events. There is sufficient evidence on effective behavior change techniques and strategies for implementation in practice (25). However, the characteristics of the target population determine the appropriate implementation strategy (33). Therefore, co-creation with end-users can provide the necessary guidance to researchers on effective implementation strategies that might not be described in published literature (34). In addition, we describe activities we did to facilitate effective co-creation of these health promotions tools.

## Materials and Methods

### Recruitment

Eligibility criteria included:

- A former or current participant of the STOP program who consented at the time of enrollment in the STOP program to be contacted for future research studies, lived in the Greater Toronto Area, and had shared at least one phone number.
- At the time of enrollment into the STOP program the participant reported at least one of the following risk factors:
  ◦ Physical inactivity - classified as being below the Canadian Physical Activity Guidelines (<150 min/week of moderate-to-vigorous activity) (35).
  ◦ Low levels of fruits and vegetable consumption - classified as being below Canada's Food Guide (2007); which recommends at least 7 servings for women and 8 servings for men per day (36).
  ◦ Risky drinking - determined by using the Alcohol Use Disorders Identification Test (AUDIT-10) (37). A score of ≥ 3 (women) and ≥4 (men) was classified as risky drinking (38).
  ◦ Low coping skills for stress - defined as a response of “Poor” or “Fine” to questions about one's ability to handle day-to-day demands and unexpected problems.
  ◦ Trouble sleeping - determined using the third item in Patient Health Questionnaire; (39, 40). A score of 1 or more on this question was classified as having trouble sleeping (40).

Eligible participants were contacted by the STOP program's research personnel once every 1–3 days until they either connected with the participant or had reached a maximum of 5 call attempts. Our goal was to recruit 22 participants for the first engagement event (Group 1) and 24 participants for the second engagement event (Group 2). These numbers were chosen given that researchers have found that a group size of 30 people or less is ideal to capture diverse experiences, allows the opportunity for all participants to contribute (41) as well as simplifying logistics (room size capacity and budget).

Participants were provided with an honorarium of $70 dollars for attending the 3 h engagement event.

### Procedure

Each engagement event was broken down into three main components.

1. A brief presentation of the scientific evidence related to the effect of modifiable risk factors on tobacco use and effective strategies for changing these risk behaviors.
2. A consensus building activity to decide the type of messages health care providers should share with their patients to communicate the need to change their behaviors. This activity followed an adapted version of the Institute of Cultural Affairs Canada (ICA) consensus building methodology (42). Thus, the following steps were performed:
  a. Brainstorm individually: The facilitator stated the purpose of the exercise; to answer the question: “*What type of a message should your practitioner tell you, in order to encourage you to make changes to some behaviors that are putting you at risk?”* Participants were given time to individually reflect on different examples of messages and brainstorm ideas. Examples of messages were adapted from existing messages that have been used by other organizations (e.g., Canadian Society for Exercise Physiology, Heart and Stroke) or research studies (43–45). As a part of their reflection, participants were also asked to choose the messages they liked or disliked.
  b. Brainstorm as a group: Participants were asked to work in small groups (2–4 participants) to share their ideas. The purpose of these discussions was not to reach agreement, but to enhance clarity of each idea. Each small group wrote their ideas on approximately 4 cards. Each card contained a single clear idea about the type of message the participant would like to receive. If two or more participants had the same idea, only one card would be written representing this idea. This way, we were able to minimize duplication yet preserve diversity.
  c. Share ideas with the larger group: The facilitator gathered the cards, read them aloud and placed them on the wall.
  d. Clustering ideas: Once 10 cards were on the wall, the facilitator asked the group to state which cards were similar, in order to form clusters. Similar cards were placed together in a column. Once a column had three cards, a symbol was placed above the three cards so that the cluster could be named without naming the idea. The facilitator continued reading the remaining cards and asked participants if each card belonged to as existing cluster or a different cluster. To allow emerging insights to evolve, participants were discouraged from naming clusters until all the cards were up on the wall.
  e. Naming the cluster: The facilitator read each of the cards in a cluster aloud and guided the group to explore the meaning behind the cluster. The group gave each cluster a name.
  f. Reflect on final product and experience: As a large group, participants were asked to reflect on what they liked and what could be improved upon.
3. An adapted Nominal Group Technique (NGT) (46) to clarify what features a self-monitoring tool(s) should include. NGT is a structured format that facilitates group brainstorming and encourages contributions from everyone. This activity involved six steps:
  a. Stating the purpose: The facilitator stated the purpose of the exercise; to answer the question: “*What type of self-monitoring tools would you like your health care provider to give you?”*
  b. Sharing examples: Different types of self-monitoring tools such as combined and individual tracking sheets were shared with the group. Participants were asked to complete each self-monitoring tool in order to acquire a more comprehensive understanding of the structure of each tracking sheet. Individual tracking sheets were defined as resources that have only one risk factor on each page. Combined tracking sheets have two or more risk factors on the same page. These resources were either taken from other organizations (i.e., American Heart Association) or were created by Picking Up the PACE.
  c. Recording ideas: Participants were asked to individually reflect and write down their approval/disapproval of each tool and brainstorm new ideas.
  d. Discussing ideas: Each participant was asked to share with the group one idea about each tracking sheet. Each new idea was recorded by the facilitator on a separate piece of flipchart paper. Participants were encouraged to ask questions to clarify any ideas that were shared.
  e. Voting on ideas: A dotmocracy system, in which participants were each given 10–12 dot stickers for voting, was conducted. Participants could place all dots on one flipchart paper (containing one idea) or distribute them across several ideas.
  f. Reflect on final product and experience: As a large group, participants were encouraged to reflect on what they liked and what could be improved upon.

Based on feedback received from the first event (Group 1), some minor modifications were made to the NGT exercise for the second event (Group 2) including: providing more time for reflection (additional 15 min), reducing the number of examples shared (from 9 examples to 6), and providing more structured questions for individual reflection such as whether they would use the resource and if so, for how long. Also, due to logistics reasons, participants in Group 1 were provided with 12 dot stickers each while those in Group 2 were provided with 10 dot stickers each.

### Analysis

Thematic analysis, “a method for identifying, analyzing and reporting patterns within data” (47) was conducted with the participants, during the events, to allow for further discussion of the findings. In the consensus building activity, the analysis phase was initiated when the generated idea cards were read aloud and displayed on the wall for the participants to view together. To ensure everyone was familiar with the ideas presented, participants were given time to examine the cards. Then, participants were asked to think critically about the similarities and differences between the ideas and to cluster similar ideas together. Thus, emergent themes were developed and participants assigned a title/label to the clusters they created. This concluded the analysis process for this activity. The final stage, reflection, was a comprehensive, all-inclusive analysis where participants reflected and shared the extent to which each title card (theme) contributed to an understanding of what the message that communicate the need for health behavior change should contain.

The second activity, which used the NGT, included a combination of qualitative and quantitative methods to elicit feedback from participants (48). The analysis phase was initiated during the large group session and a thematic approach was used to determine participants' preferred way of self-monitoring their risk behaviors. Participants were first asked to reflect individually and then, as a group, and share what they liked or disliked about the self-monitoring resources as well as express any additional features that should be considered. These suggestions (themes) were placed on the wall and participants were given time to review and compare the different themes. Participants were then asked to vote on each theme. The results of the voting became the main outcome of interest for the activity. The written responses from the individual reflection section of this activity were reviewed post-event to provide additional context to the final results.

## Results

### Participants

Nine of 22 invited participants agreed to participate in Group 1, while 18 of the 24 invited participants agreed to participate in Group 2. Unexpected hospital and family commitments were the primary reasons given by patients for not attending the engagement events. The demographic information for both groups is presented in Table 1. Most participants (93%) had a household income of <$40,000 (CAD), which is below the median income in Canada. This is considered to be low income for a four person household (49, 50). Low socioeconomic status has been associated with a greater likelihood of having modifiable risk factors (51–53) and presents additional barriers to successful behavior change (54–56). As a result, representation from this population provides an opportunity to better understand how interventions need to be tailored and delivered.

**Table 1:** Demographic information of participants who attended the events^†^.

**Variables**	**Group 1**	**Group 2**
**Individual-level**	**(*****n*** **=** **9)**	**(*****n*** **=** **18)**
Age in years (mean, sd)	56.7 (9.3)	53.1 (12.5)
Male, *n* (%)	5 (56%)	10 (56%)
High school diploma or higher, *n* (%)	4 (44%)	17 (94%)
Household income above 40 k, *n* (%)	0 (0%)	2 (11%)
Currently employed, *n* (%)	2 (22%)	6 (33%)
Daily smokers, *n* (%)	8 (89%)	17 (94%)
Proportion of participants who have quit at least once in the past year, *n* (%)	5 (56%)	9 (50%)
Importance of quitting rating (mean, sd)	8.6 (2.2)	9.4 (0.8)
Confidence in quitting smoking rating (mean, sd)	8.5 (1.7)	6.9 (2.7)
Proportion of participants with at least one physical comorbid condition^‡^, *n* (%)	5 (56%)	5 (28%)
Proportion of participants with at least one psychiatric comorbid condition^§^, *n* (%)	7 (78%)	13 (72%)
Proportion of participants with substance use disorder^¶^, *n* (%)	3 (33%)	5 (28%)
**Organization type**		
Family Health Team, *n* (%)	3 (33%)	6 (33%)
Community Health Centre, *n* (%)	6 (67%)	10 (56%)
Addiction Agency, *n* (%)	0 (0%)	2 (11%)

† *The sum of percentages may not equal 100% due to rounding*.

‡ *Physical comorbid conditions include heart disease, stroke, diabetes, chronic obstructive pulmonary disorder, rheumatoid arthritis, cancer*.

§ *Psychiatric comorbid conditions include depression, anxiety, schizophrenia, bipolar disorder*.

¶ *Excludes tobacco and caffeine*.

### Consensus Building Activity – Messages Focusing on Need to Change Health Behaviors

Participants were asked to brainstorm and generate ideas about the types of messages they wanted health practitioners to convey to their patients. Group 1 and Group 2 generated 14 and 19 ideas, respectively. Facilitators NM and ML guided participants to cluster the ideas into groups and to categorize each cluster. By the end of the exercise, Group 1 created five clusters and Group 2 created three clusters. Group 2 was not aware of the clusters that Group 1 had created. The categories that groups had chosen for their respective clusters can be found in Table 2.

**Table 2:** Cluster categorization from the consensus building activity.

**Question: What type of a message should your practitioner tell you; in order to encourage you to make changes to your risk factor?**	
**Group 1**	**Group 2**
Positive Reinforcement	Positive Compassion. Emphasis on Mental and Emotional Well-being
Pro-active/Never a Failure/ Positive Affirmation	Encouraging Practitioners to be Aware of Patient's Circumstances and Resources
Empowering with More Information	Strategies for Patients/Use Psychological Techniques and Raise Awareness
Reality Check	
Have Visuals to Help Your Patient	

Participants from both groups created categories which reflect their preference of practitioners communicating messages that provide positive reinforcement, affirmation and compassion. They also recommended empowering patients with more information and raising awareness. Group 1 preferred practitioners to also provide patients with reality checks (comments that would make patients recognize the risks of their unhealthy behaviors), while Group 2 wanted practitioners to be more aware of the patients' circumstances.

### NGT – Self-Monitoring Resources

Both groups were provided examples of two types of self-monitoring resources: individual and combined tracking sheets. After allowing for individual reflection, participants were asked to share ideas about which self-monitoring resources they preferred and what should be included in a tracking sheet. Each new idea was recorded on separate flip chart papers. At the end of the discussion, participants were asked to vote using dot stickers on the idea(s) they preferred the most. In Group 1, each participant was given 12 dot stickers and was allowed to vote on more than one idea. Table 3A shows all the ideas that Group 1 generated and is ordered from highest to lowest by the number of votes. This same exercise was repeated with Group 2. Due to logistical reasons, participants in Group 2 were provided with 10 dot stickers each. The results of Group 2's dotmocracy can be found in Table 3B.

**Table 3A:** Group 1's dotmocracy results for the types of self-monitoring resources (tracking sheets).

**Ideas**	**Vote**
Combined Tracking Sheet with More Room (Example F)	22
Apps	15
Easy and Simple Alcohol Tracker (Example D)	13
Multi-Risk Factor Tracking Sheet (Example B)	12
Smoking and Mood Biweekly Tracking Sheet (Example E)	9
Sleep Diary Tracker (Example G)	6
Separate/Individual Trackers	4

**Table 3B:** Group 2's dotmocracy results for the types of self-monitoring resources (tracking sheets).

**Ideas**	**Vote**
Multi-Risk Factor Tracking Sheet (Example B)	55
Smoking and Mood Biweekly Tracking Sheet (Example E)	28
Physical Activity Tracking Sheet (Example A)	23
Alcohol Only Tracking Log (Example D)	21
Physical Activity and Healthy Eating Tracking Sheet (Example F)	11
Smoking, Alcohol, and Mood Tracking Sheet (Example C)	6
Apps	0
Able to Journal and Explain Thoughts/Events	0
More Information About Just One Behavior	0
Offer More Than One Type of Tracking Sheet so Patients Have a Choice	0

## Discussion

During the two engagement events, participants expressed a preference for healthcare practitioners to provide health behavior messages that included positive reinforcement, compassion, and affirmation. Participants discussed a greater need for information that would provide empowerment and allow the patient to participate in any decisions regarding prevention or treatment. The majority of participants preferred to monitor their behavioral changes through a single page self-reported tracking sheet that included all of the risk behaviors that the participant was attempting to change. Participants perceived the Multiple Risk Factor Tracking Sheet as being simple, efficient, informative, and provided an opportunity to make connections between risk behaviors. Furthermore, the majority of participants who selected the Multiple Risk Factor Tracking Sheet expressed interest in using it for at least a 1-month period. Such compliance with completing the Multiple Risk Factor Tracking Sheet would provide greater insight in the behavioral change process. Other tracking sheets offered to participants were perceived to be time consuming and too long.

Integrating patients into the planning and delivery of health care requires effective communication between both parties; patients and health care practitioners (57). When communicating to patients, practitioners can either choose to frame health information by emphasizing the attainment of beneficial or positive outcomes (gain-framed messages) or the costs or negative outcomes (loss-framed messages) (58, 59). The findings from this study encourage the use of gain-framed messages when discussing behavioral changes with participants. This is consistent with research published in recent years demonstrating gain-framed messages to be effective at promoting physical activity (60), intentions to consume adequate quantities of fruits and vegetables (61), and smoking cessation (62).

This study outlines a methodology that can alleviate some of the tensions that exist between two key values in health promotion—namely, evidence-based approaches to population health and public and patient engagement (63). Participants provided valuable insight into the wording and structure of messages. Furthermore, participants co-developed a self-monitoring resource that is simple and efficient. Due to participant involvement in the creation of these tools, it is expected that uptake of the tools will be enhanced as participants will be more receptive to messages that communicate the need for health behavior change and more likely to complete the self-monitoring resource. The intent of this study was not to provide information on the adaptability or effects of messaging and self-monitoring techniques on behavioral change, but rather to inform the messages and self-monitoring tools that STOP health care providers could use with their patients. These resources are currently being used in the STOP program and are part of an Ontario wide study, Picking Up the PACE (64). Future research in the field of co-creation should consider examining how co-created materials impact the effectiveness of interventions.

This study has some limitations. First, participants were required to attend each engagement event in-person. Consequently, our sample of participants was restricted to individuals residing in the Central Toronto area. This may have potentially created a selection bias in which the views and opinions of the recruited participants may not have been representative of those individuals residing throughout Ontario. Furthermore, since the invitation for the engagement event was distributed by the Center for Addiction and Mental Health, the institution that implements the STOP program, there is a risk of social desirability bias. Participants may have felt compelled to provide responses that may appear more favorable to the facilitators. That being said, none of the facilitators were known to the participants prior to the event and had no role in their clinical care. Given that 41% of invited participants did not attend the engagement events, an additional limitation is the inclusion of possible response bias. However, the feedback provided by the participants who attended (e.g., positive messages) coincides with existing literature that shows gain-framed messages are effective for health behavior change (60–62). Moreover, we hosted two separate engagement events with separate groups of people and the feedback from both groups was very similar. Given the consistency in the results, we believe we achieved saturation using this methodology. While our sample of participants were primarily older, this is quite representative of the target population of the intervention as the average age of participants in the STOP program is 52 years old.

## Conclusions

The results of these engagement events will be used to inform the design of an online tool developed by the PACE initiative to help guide practitioners with addressing multiple health behaviors in the STOP program. We will develop health behavior messages that communicate positive messages and these messages will be incorporated into our tool. As well, the Multiple Risk Factor Tracking Sheet will be adapted and incorporated into the PACE intervention for practitioners to offer to their patients in supporting their efforts in monitoring their behaviors.

## Data Availability Statement

The raw data supporting the conclusions of this article will be made available by the authors, upon reasonable request.

## Ethics Statement

The studies involving human participants were reviewed and approved by The Center for Addiction and Mental Health. The patients/participants provided their written informed consent to participate in this study.

## Author Contributions

NM, ML, and PS developed the original concept of this study. NM and ML conducted the patient engagement event. NM, ML, and WR wrote the first draft of the research paper. All authors participated in the critical revision of the manuscript, contributed to the study design, read and approved the final manuscript.

## Conflict of Interest

PS reports receiving grants and/or salary and/or research support from Centre for Addiction and Mental Health, Health Canada, Ontario Ministry of Health and Long-Term care, CIHR, Canadian Centre on Substance Use and Addiction, Public Health Agency of Canada, Medical Psychiatry Alliance, Canadian Cancer Society Research Institute, Cancer Care Ontario, and the Ontario Institute for Cancer Research, McLaughlin Centre, Academic Health Sciences Centre, Workplace Safety and Insurance Board, National Institutes of Health, The Association of Faculties of Medicine of Canada. PS also reports receiving funding from the following commercial organizations Pfizer Inc./Pfizer Canada, Bhasin Consulting Fund, Shoppers Drug Mart and Patient-Centered Outcomes Research Institute, ABBVie, Bristol-Myers Squibb; and has received consulting fees from Pfizer Inc./Pfizer Canada, Johnson & Johnson Group of Companies, MedPlan Communications, Evidera Inc., Kataka Medical Communications, Miller Medical Communications, Nvision, Insight Group, Sun Life Financial, Inflexxion Inc. Through an open tender process, Johnson & Johnson, Novartis, and Pfizer Inc. are vendors of record for providing smoking cessation pharmacotherapy, free or discounted, for research studies in which PS is the principal investigator or co-investigator. NM receives salary from the Centre for Addiction and Mental Health and has received grants from Canadian Institutes of Health Research, Canadian Cancer Society Research Institute, Public Health Agency of Canada, and Health Canada. WR reports grant funding from Pfizer Inc., and Public Health Agency of Canada. WR also reports stock ownership in Abbott Laboratories. None of these companies had any involvement in the study design, data collection/analysis, decision to publish, or preparation of this manuscript. The remaining authors declare that the research was conducted in the absence of any commercial or financial relationships that could be construed as a potential conflict of interest.

## Abbreviations

AUDIT-10: Alcohol Use Disorder Identification Test
PHAC: Public Health Agency of Canada
ICA: Institute of Cultural Affairs Canada
MPA: Medical Psychiatry Alliance
NGT: Nominal Group Technique
PACE: Promoting and Accelerating Change through Empowerment
STOP: Smoking Treatment for Ontario Patients.
